# Gold-Film-Thickness Dependent SPR Refractive Index and Temperature Sensing with Hetero-Core Optical Fiber Structure

**DOI:** 10.3390/s19194345

**Published:** 2019-10-08

**Authors:** Rui Zhang, Shengli Pu, Xinjie Li

**Affiliations:** 1 College of Science, University of Shanghai for Science and Technology, Shanghai 200093, China; 2 Shanghai Key Laboratory of Modern Optical System, University of Shanghai for Science and Technology, Shanghai 200093, China

**Keywords:** refractive index sensing, temperature sensing, optical fiber sensor, hetero-core fiber structure, surface plasmon resonance

## Abstract

A simple hetero-core optical fiber (MMF-NCF-MMF) surface plasmon resonance (SPR) sensing structure was proposed. The SPR spectral sensitivity, full width of half peak (FWHM), valley depth (VD), and figure of merit (FOM) were defined to evaluate the sensing performance comprehensively. The effect of gold film thickness on the refractive index and temperature sensing performance was studied experimentally. The optimum gold film thickness was found. The maximum sensitivities for refractive index and temperature measurement were obtained to be 2933.25 nm/RIU and −0.91973 nm/°C, respectively. The experimental results are helpful to design the SPR structure with improved sensing performance. The proposed SPR sensing structure has the advantages of simple structure, easy implementation, and good robustness, which implies a broad application prospect.

## 1. Introduction

Surface plasmon resonance (SPR) optical fiber sensor has the advantages of ultra-high sensitivity and resolution, fast response, small size, and real-time detection capability, which has been widely studied in environmental monitoring [1,2], disease diagnosis [3,4], biological analysis [5,6], and other fields [7,8]. Its sensing performance mainly depends on the geometry and structure parameters of optical fiber, type of metal coating, etc. In order to increase the evanescent wave outside the optical fiber, removing some or all of the optical fiber cladding is employed with the traditional SPR optical fiber sensor. For example, the typically processed fiber structures include etched fibers [9,10], side polished fibers (SPF) [11,12], tapered fibers [13,14], D-shaped fibers [15,16], etc. Through this way, the SPR effect is enhanced. However, removal of cladding will reduce the structural strength of the fibers and result in decline in mechanical properties. To avoid the damage of fiber structure, high sensitivity SPR optical sensors can also be realized by using grating structure, such as tilted fiber Bragg grating (TFBG) [17,18], fiber Bragg grating (FBG) [19,20], and long period fiber grating (LPG) [21,22]. These grating structures maintain the integrity of the optical fiber and are easy to operate, but the fabrication cost is high.

The mentioned problems can be solved with hetero-core fiber structure [23,24,25], which possesses the merits of simple structure, low cost, high sensitivity, and good mechanical performance. Therefore, we fabricated simple hetero-core optical fiber (MMF-NCF-MMF) structures using coreless optical fibers (NCF) as sensing regions. Due to the absence of cladding, the core mode of NCF can directly contact with the external environment. When light propagates from the lead-in multimode optical fiber (MMF) to NCF, evanescent wave outside the NCF can resonate with surface plasma wave (SPW) without any complicated processing. On the other hand, silver or gold is usually used as the metal material to excite SPR. But silver is easier to oxidize than gold. So gold is more often used as the coating material. The performance of gold-coated fiber SPR sensor is closely related with the coating thickness.

In this work, the MMF-NCF-MMF hetero-core fiber structure is coated with a gold thin film and the influence of gold film thickness on the refractive index and temperature sensing performance of the as-fabricated SPR sensing structure is investigated.

## 2. Sensor Fabrication and Sensing Principle

**Figure 1:**
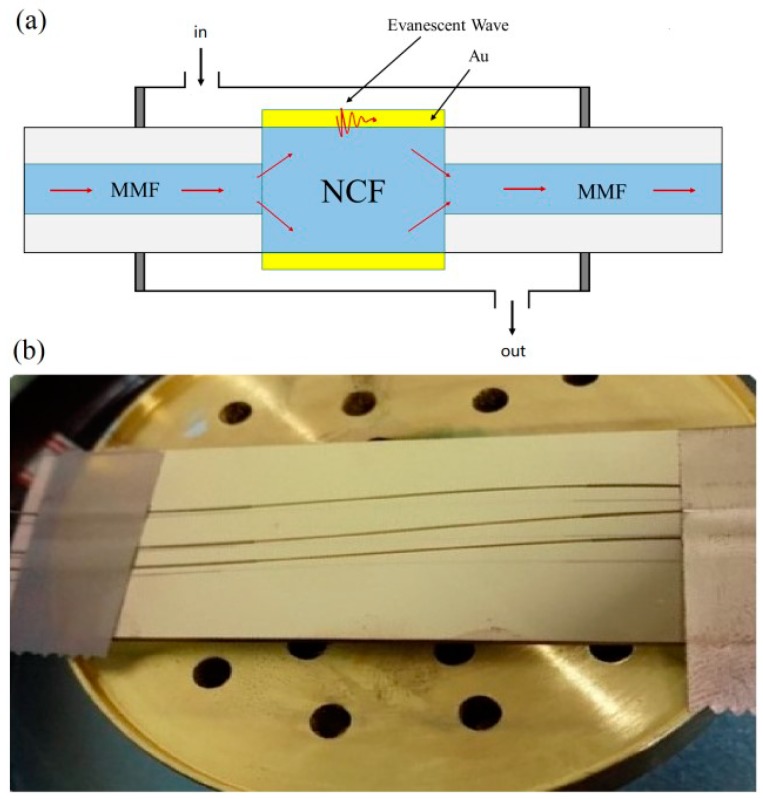
(**a**) Schematic of the proposed sensor; (**b**) Picture of the as-fabricated structure coated with gold film.

The sensor structure is schematically shown in Figure 1a. The NCF with a length of 1 cm is spliced between two MMFs. The core/cladding diameters of the MMF and NCF are 62.5/125 μm and 0/125 μm, respectively. Gold thin film is coated on the NCF by magnetron sputtering device (ETD-900M, Elaborate Technology Development, China). The process of coating is simplified by double-sided coating method [26]. Figure 1b is the picture of the as-fabricated structure coated with gold film.

**Figure 2:**
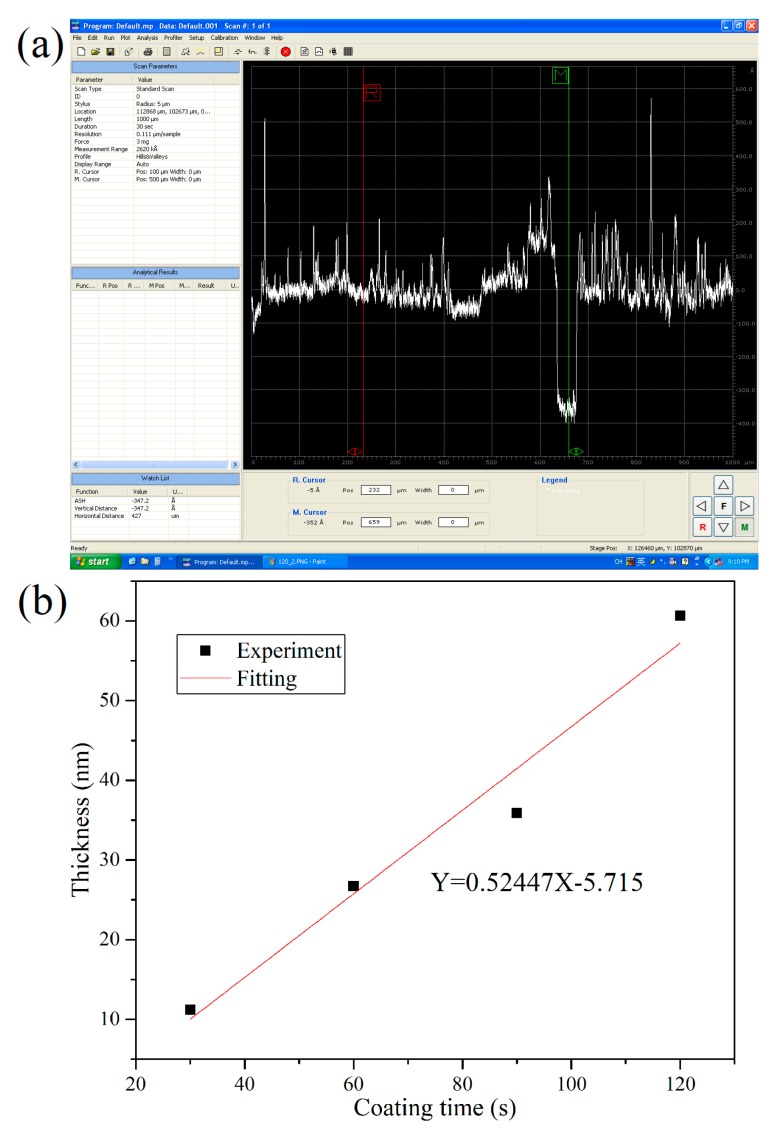
(**a**) Typical screenshot of the step profiler for measuring the gold film thickness with sputtering time of 90 s; (**b**) Gold film thickness as a function of sputtering time.

The gold film thicknesses are measured by a step profiler. The typical screenshot of the step profiler for measuring the gold film thickness with sputtering time of 90 s is shown in Figure 2a. Figure 2b shows the measured gold film thickness as a function of the sputtering time, which implies that the gold film thickness increases linearly with the sputtering time. Hereafter, the gold film thickness for other samples with other different sputtering time is estimated according to Figure 2b.

The glycerol aqueous solution and ethanol are utilized for the refractive index and temperature sensing experiments, respectively. Ethanol has a relatively high thermo-optical coefficient of around −4 × 10^−4^ RIU/°C, which is two orders of magnitude higher than that of silica, which is about 9.2 × 10^−6^ RIU/°C [27,28].

There are several conditions to excite the optical fiber SPR sensing structure: (1) phase matching; (2) polarization of light is perpendicular to the metal surface; (3) sensing region is in the range of evanescent field. The evanescent field is usually only 100−200 nm in depth and decays exponentially away from the interface between the fiber and gold film. Theoretically, the penetration depth d of evanescent field is related to incident angle *θ*, refractive index of fiber core and cladding, which can be expressed as [1]:

(1) $$d=\frac{\lambda }{2\pi \sqrt{n_{1}^{2}\sin^{2}\theta -n_{2}^{2}}},$$

where *n*_1_ and *n*_2_ are the refractive indices of fiber core and cladding, respectively. *λ* is the incident wavelength.

The propagation constant of surface plasmon polaritons (SPP) is expressed as [5,29]:

(2) $$\beta_{SPP}=\frac{\omega }{c}\sqrt{\frac{\varepsilon_{m}\varepsilon_{s}}{\varepsilon_{m}+\varepsilon_{s}}},$$

where c is the speed of light in vacuum, *ω* is the angular frequency of light, $\varepsilon_{m}$ and $\varepsilon_{s}$ are the relative dielectric constants of gold film and surrounding environment near the metal surface, respectively. Therefore, the phase-matching condition can be expressed as:

(3) $$\beta_{i}=\beta_{SPP},$$

where *β*_i_ is the propagation constant of the fiber mode, i is the order number of the fiber mode. According to Equations (2) and (3), the resonance wavelength will change when the refractive index (or dielectric constant) of the surrounding environment changes. Therefore, the sensing function can be realized.

## 3. Experiments Results and Discussion

**Figure 3:**
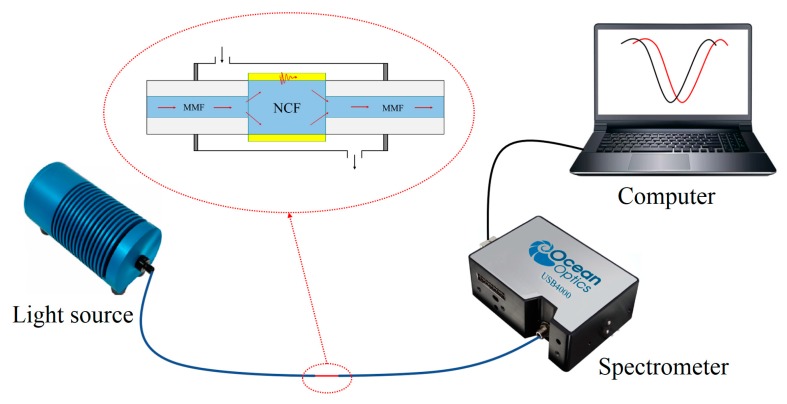
Experimental setup for investigating the sensing properties of the simple hetero-core optical fiber (MMF-NCF-MMF) structure.

The experimental setup for investigating the sensing performance of the as-fabricated hetero-core fiber structure is shown in Figure 3. The sensing system mainly includes halogen light source (360–2000 nm, HL-2000, Ocean Optics (Shanghai) Co., LTD, China), sensing part, optical fiber spectrometer (USB4000, Ocean Optics (Shanghai) Co., LTD, China), and computer. An optical fiber spectrometer is used to collect the output spectrum, which is further recorded and analyzed by the computer.

**Figure 4:**
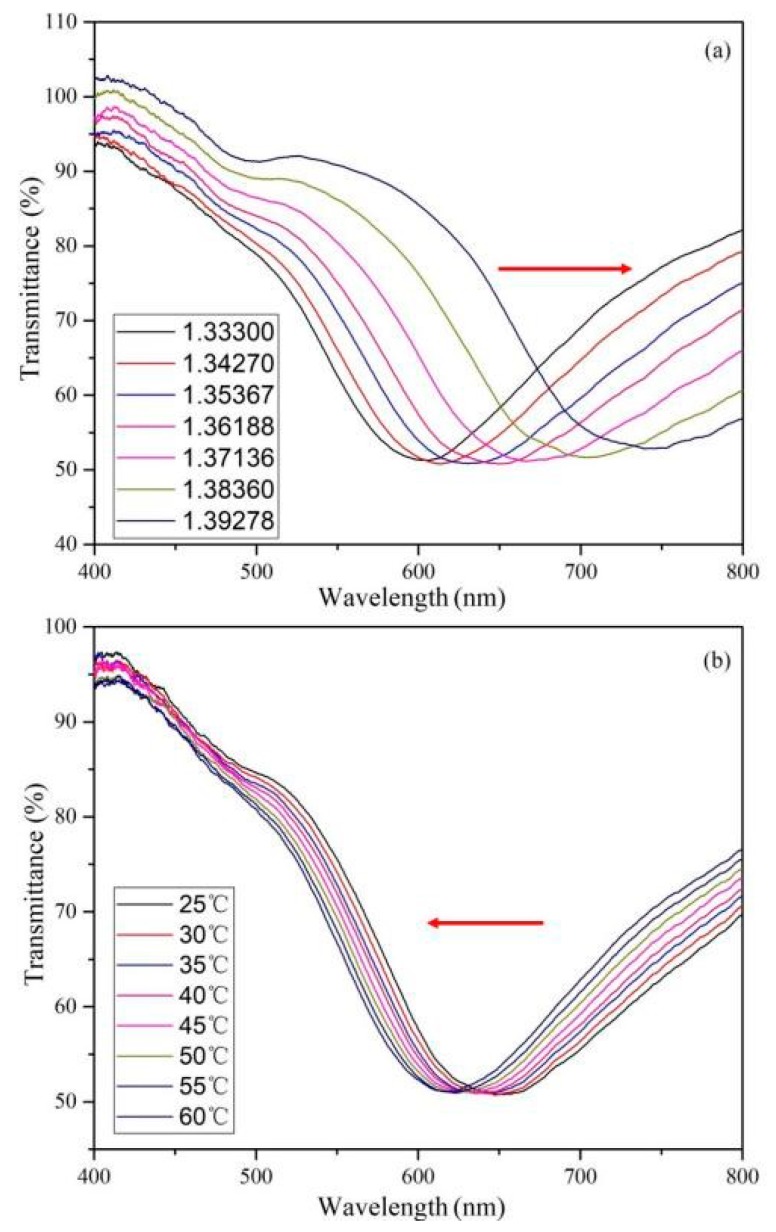
Transmission spectra of the sensing structure at different surrounding refractive indices (**a**) and ambient temperatures (**b**). The thickness of the gold film is 25.753 nm.

Figure 4 shows the transmission spectra of the sensing structure at different surrounding refractive indices and ambient temperatures, respectively. The thickness of the coated gold film is 25.753 nm. The measured SPR spectrum is normalized according to the following definition:

(4) $$P_{\mathrm{trans}}=\frac{T_{\mathrm{sol}}-D_{bg}}{T_{\mathrm{air}}-D_{\mathrm{bg}}},$$

where $T_{\mathrm{sol}}$ and $T_{\mathrm{air}}$ are the measured intensity when the structure is in measurand and air, respectively. $D_{bg}$ is the background signal. To reduce high-frequency noise, each spectrum has been smoothed. Figure 4 shows that the resonance wavelength drifts to a long wavelength with the external refractive index. However, the resonance wavelength drifts to a short wavelength with the increase of temperature, which is due to the decrease of the refractive index of ethanol solution at high temperature. On the other hand, the intensity of the SPR wavelength dip remains unchanged, which reflects the measurement stability of the sensing structure.

**Figure 5:**
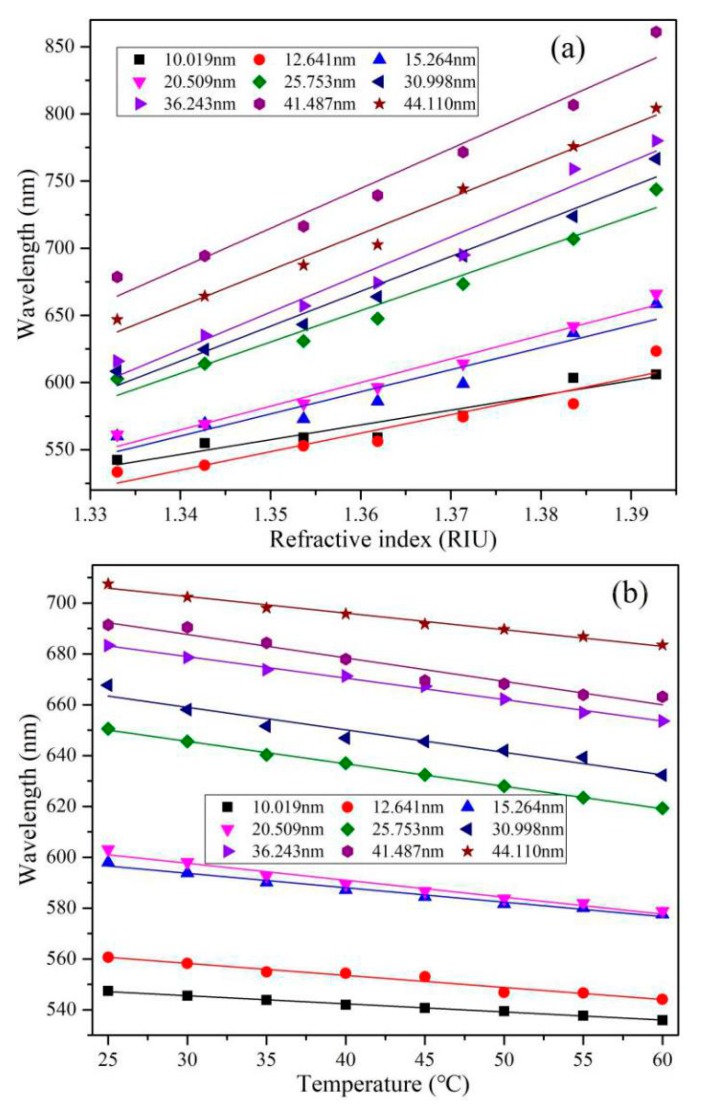
Resonance wavelength as a function of surrounding refractive index (**a**) and ambient temperature (**b**) at different gold film thicknesses.

Similarly, the refractive-index and temperature-dependent spectra for other samples with different gold film thicknesses are obtained. Figure 5 displays the variation of resonance wavelength with surrounding refractive index and ambient temperature at different gold film thicknesses, respectively. The higher the external refractive index is, the larger the resonance wavelength is. Contrarily, the resonance wavelength shifts to a short wavelength with the increase of temperature, which is due to the negative thermo-optical coefficient of ethanol. At fixed external refractive index or ambient temperature, the resonance wavelength drifts to a long wavelength with gold film thickness. Some experimental data deviate from the linear fitting, which may be due to the errors in film thickness, refractive index, and temperature [30].

**Figure 6:**
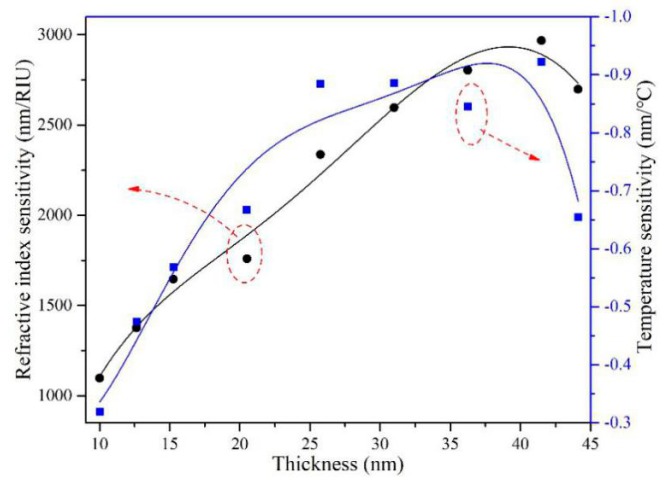
Refractive index and temperature sensitivities as functions of gold film thickness.

Figure 6 shows the refractive index and temperature sensitivities as functions of gold film thickness. The polynomial fitting method was employed. It can be seen from Figure 6 that both sensitivities increase first and then decrease with the increase of film thickness. When the thickness of gold film is around 40 nm, the refractive index and temperature sensitivities are the highest, which are 2933.25 nm/RIU and −0.91973 nm/°C, respectively.

**Figure 7:**
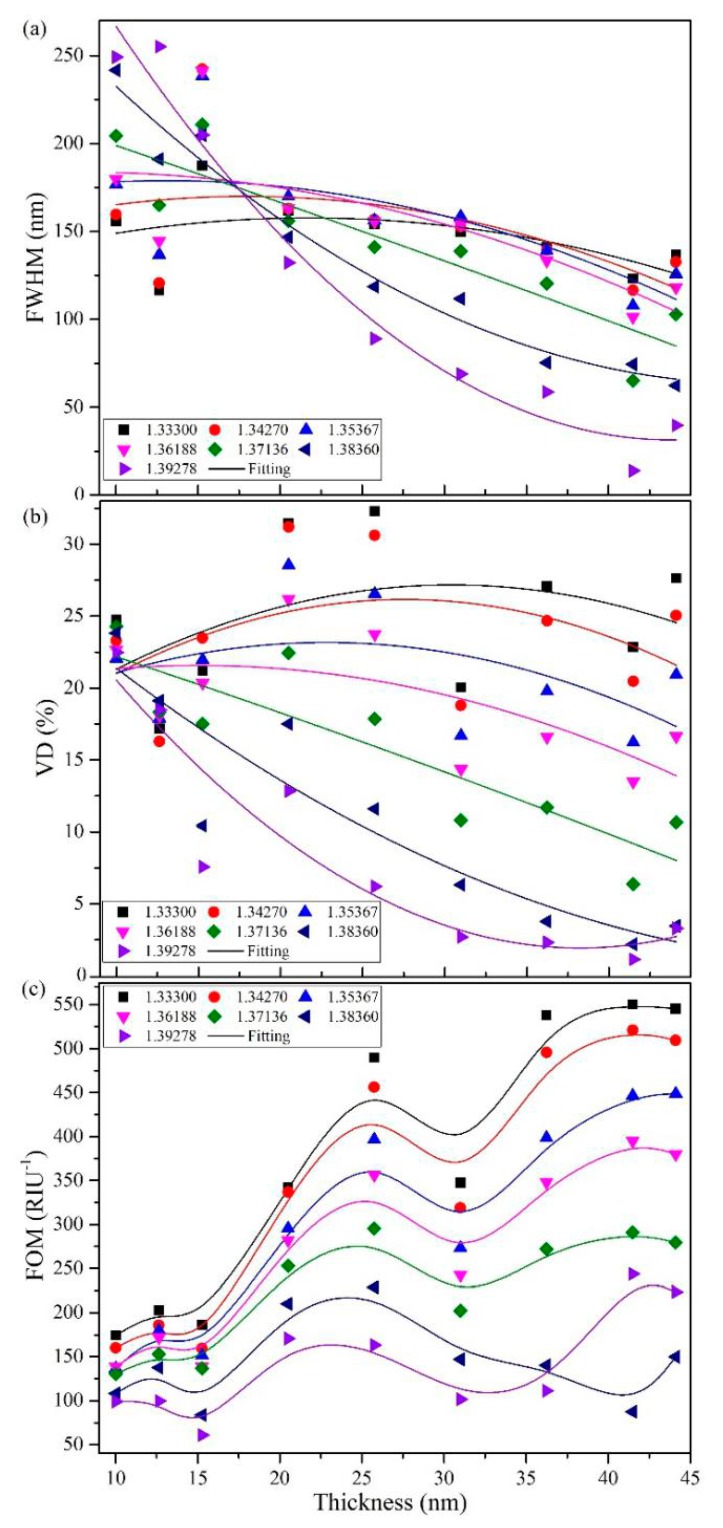
Gold film thickness dependence of (**a**) full width of half peak (FWHM), (**b**) valley depth (VD), and (**c**) figure of merit (FOM) during refractive index measurement.

In order to further characterize the effect of gold film thickness on SPR spectra, full width of half peak (FWHM), valley depth (VD), and figure of merit (FOM) of the SPR spectra are analyzed. The FOM is defined as $FOM=\frac{S}{FWHM}\times VD$, where S is the sensitivity of the sensor. VD is defined as the transmittance difference between the maximum (on the lower side) and minimum values forming the corresponding valley. Figure 7 shows the gold-film-thickness dependent FWHM, VD, and FOM of the sensing structure at different refractive indices (at an ambient temperature of 20 °C).

Figure 7a indicates FWHM decreases with the increase of film thickness for certain external refractive index. Besides, the larger the external refractive index is, the larger the change of FWHM with the film thickness will be. However, FWHM increases with the increase of the refractive index when the film thickness is less than 18 nm. When the thickness is greater than 35 nm, FWHM decreases with the increase of refractive index. When the film thickness is between 18 and 35 nm, FWHM increases first and then decreases with the refractive index.

Figure 7b shows that VD increases with film thickness at the small refractive index regime. When the external refractive index is large, VD decreases with the film thickness. For samples with certain film thickness, the VD decreases with the increase of refractive index.

Figure 7c shows that FOM increases with the gold film thickness in general. In most cases, the largest FOM is achieved for the sample with a film thickness of 41.487 nm. Therefore, the sensor with the best performance can be obtained for the sample with a gold film thickness of 41.487 nm.

**Figure 8:**
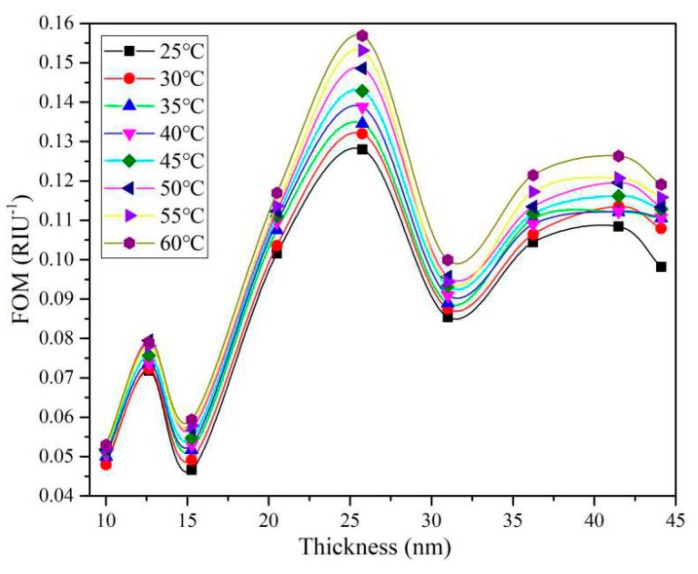
Gold film thickness dependence of FOM during temperature measurement.

Similarly, the gold-film-thickness dependent FOM of the sensing structure at different ambient temperatures (at an initial refractive index of 1.361) is calculated and plotted in Figure 8. It can be seen from Figure 8 that the optimum gold film thickness is around 25.753 nm. This result guides us to optimize the gold film thickness reasonably to make the sensor have the best performance for different application cases.

## 4. Conclusions

The dependence of sensing performance of the MMF-NCF-MMF hetero-core fiber SPR structure on the gold film thickness was studied experimentally. The corresponding refractive index and temperature sensitivities are 2933.25 nm/RIU and −0.91973 nm/°C, respectively. The variation of FWHM, VD, and FOM with refractive index and temperature are discussed and analyzed. The results show that the proposed sensor has different optimal gold film thicknesses for temperature and refractive index measurements. The proposed SPR sensing structure has the features of simple structure, easy implementation and good robustness, which has great application prospect.
